# Trends in the Utilization of *BRCA1* and *BRCA2* Testing After the Introduction of a Publicly Funded Genetic Testing Program

**DOI:** 10.3390/curroncol32080439

**Published:** 2025-08-06

**Authors:** Fahima Dossa, Nancy N. Baxter, Rinku Sutradhar, Tari Little, Lea Velsher, Jordan Lerner-Ellis, Andrea Eisen, Kelly Metcalfe

**Affiliations:** 1 Department of Surgery, Memorial Sloan Kettering Cancer Center, New York, NY 10065, USA; 2 Faculty of Medicine and Health, University of Sydney, Sydney, NSW 2006, Australia; nancy.baxter@sydney.edu.au; 3 Institute of Health Policy, Management, and Evaluation, University of Toronto, Toronto, ON M5S 1A1, Canada; rinku.sutradhar@ices.on.ca; 4 ICES, Toronto, ON M4N 3M5, Canada; 5 Li Ka Shing Knowledge Institute, Unity Health, Toronto, ON M5C 2T2, Canada; 6 Division of Biostatistics, Dalla Lana School of Public Health, University of Toronto, Toronto, ON M5S 1A1, Canada; 7 Medical Genetics, London Health Sciences Centre, London, ON N6A 5W9, Canada; tari.little@lhsc.on.ca; 8 Genetics Program, North York General Hospital, Toronto, ON M2K 1E1, Canada; lvelsher@nygh.on.ca; 9 Laboratory Medicine and Pathobiology, University of Toronto, Toronto, ON M5S 1A1, Canada; jordan.lerner-ellis@sinaihealth.ca; 10 Lunenfeld-Tanenbaum Research Institute, Sinai Health System, Toronto, ON M5G 1X5, Canada; 11 Pathology and Laboratory Medicine, Mount Sinai Hospital, Sinai Health System, Toronto, ON M5G 1X5, Canada; 12 Odette Cancer Centre, Sunnybrook Health Sciences Centre, Toronto, ON M4N 3M5, Canada; andrea.eisen@sunnybrook.ca; 13 Lawrence S. Bloomberg Faculty of Nursing, University of Toronto, Toronto, ON M5S 1A1, Canada; kelly.metcalfe@utoronto.ca; 14 Women’s College Research Institute, Toronto, ON M5S 1B2, Canada

**Keywords:** breast cancer, genetic testing, cancer prevention

## Abstract

Purpose: To effectively reduce cancer burden, genetic testing programs should identify high-risk individuals prior to cancer development, when risk-reduction strategies can be implemented. We evaluated trends in *BRCA1*/*BRCA2* testing use after implementation of a publicly funded testing program. Methods: We conducted a retrospective, near population-based study of women who underwent *BRCA1*/*BRCA2* testing in Ontario, Canada, (2007–2016) (*n* = 15,986). Temporal trends were evaluated using linear and Poisson regression. Results: Although annual utilization of testing increased over time (*p* < 0.001), mean age at testing increased from 49.9 years (SD 13.8) in 2007 to 53.8 years (SD 13.7) in 2016 (*p* < 0.001). The proportion of women with a cancer history at testing also increased from 53.5% in 2007 to 66.3% in 2015 (*p* < 0.001); the proportion of women free from breast cancer did not change significantly (49.2% in 2007 versus 45.1% in 2015, *p* = 0.90). As a proportion of all tested, those with breast cancer tested within 3 months of diagnosis increased over time (0.39% of tests in 2007 versus 13.6% of tests in 2015; *p* < 0.001). Conclusions: While the institution of a publicly funded genetic testing program was associated with rising utilization, increasing age at testing and decreasing testing of unaffected women suggest limitations in identifying high-risk individuals eligible for risk-reduction.

## Simple Summary

When utilized effectively, genetic testing for cancer risk-increasing mutations, (such as pathogenic mutations in the *BRCA1* and *BRCA2* genes) can identify high-risk individuals prior to cancer development, allowing for tailored interventions aimed at early cancer detection and prevention. In this study of trends in *BRCA1* and *BRCA2* testing after the implementation of a publicly funded testing program in Ontario, Canada, we demonstrate increasing utilization of testing over time. However, we find that testing largely focused on women with cancer and that testing has not increased among cancer-free women who stand to gain the most from cancer risk-reducing interventions. With greater accessibility to germline genetic testing, this study highlights the need for targeted strategies to identify and test high-risk individuals before cancer development.

## 1. Introduction

Individuals with pathogenic variants in the *BRCA1* and/or *BRCA2* genes face elevated risks of cancer, including a 70% lifetime risk of breast cancer and 40% lifetime risk of ovarian cancer [1]. If detected prior to cancer development, the risk of developing cancer may be reduced by risk-reducing surgery or chemoprevention. When a pathogenic variant is identified at the time of cancer diagnosis, adjuvant treatments can be tailored, e.g., use of PARP-inhibitors [2,3,4]. Additionally, knowledge of *BRCA1* or *BRCA2* variant carriers can inform testing of family members (i.e., cascade testing). *BRCA1* and *BRCA2* testing, therefore, plays a crucial role in guiding clinical management, risk reduction, and personalized treatment plans.

The ideal genetic testing program would serve multiple aims. First, identify high-risk individuals before cancer development, including through cascade testing, to facilitate uptake of risk-reduction strategies to reduce cancer burden. Furthermore, in those who develop cancer prior to knowledge of variant status, testing would be offered close to the time of diagnosis to guide treatments. Ideally, over time, more unaffected individuals would be tested at ages prior to when cancers would be expected to develop so efforts could be focused on cancer risk-reduction options and intensified screening. Despite the clinical availability of *BRCA1* and *BRCA2* testing in the US since 1996, studies show suboptimal uptake, with variation in utilization by socioeconomic status, race and ethnicity, and geography [5,6,7,8,9,10]. Barriers include lack of patient awareness of eligibility for testing, lack of physician awareness, insufficient counseling capacity, failure to communicate variant status with family members, cost, and access to testing [11,12,13].

In the early 2000s, the province of Ontario, Canada, expanded access to testing by implementing a publicly funded *BRCA1/BRCA2* genetic testing program for any individual who meets one of thirteen high-risk criteria [14]. By reducing reliance on cost and access, such programs can potentially decrease disparities in testing; however, it is not known whether these programs achieve the goals identified above. This information is particularly germane with the recent broad adoption of genetic panel testing. We have collected genetic testing data for women who underwent *BRCA1* and *BRCA2* testing in Ontario, Canada, as part of the *What Comes Next Cohort* [14,15]. The aim of this study was to evaluate trends in utilization of *BRCA1* and *BRCA2* testing in Ontario since the inception of this program and to identify gaps in testing in order to inform targeted strategies for cancer risk reduction.

## 2. Materials and Methods

### 2.1. Study Design and Participants

The protocol for development of the *What Comes Next Cohort* has previously been described [15]. We identified adult women (≥18 years), with or without a cancer diagnosis, who underwent *BRCA1* and/or *BRCA2* testing between 1 January 2007 and 30 April 2016 at two regional genetic testing labs: Mount Sinai Hospital (MSH), Toronto, and North York General Hospital (NYGH). Together, these sites perform approximately 70% of *BRCA1/BRCA2* testing in Ontario. We performed chart abstraction to obtain demographic, family history, and genetic testing information. We deterministically linked records to administrative health databases housed at ICES, an independent, non-profit research institute that collects and analyzes healthcare and demographic data for health system evaluation and improvement. Datasets were linked using unique encoded identifiers and analyzed at ICES.

This study received ethics approval from the Research Ethics Boards at MSH, NYGH, Sunnybrook Health Sciences Centre, and the University of Toronto.

### 2.2. Chart Abstraction

We used patient charts at MSH and NYGH to obtain data on genetic testing and ancestry of cohort participants. From genetics charts, we obtained dates and indications for testing, test type, and result. Genetic testing type was categorized as predictive/familial testing (for a specific gene variant known to be carried by a family member), Ashkenazi founder testing (testing for the three variants carried in highest frequency among the Ashkenazi Jewish population), and complete analysis (sequencing of the coding region of the *BRCA1* and *BRCA2* genes and splice sites, + or − 15 base pairs from the exon junction, as well as deletion duplication analysis). For the purposes of this study, test types are reported as Predictive (familial) and Non-Predictive (founder testing and complete analysis). We determined ancestry of participants from pedigrees submitted by genetic counselors prior to genetic testing.

### 2.3. Administrative Record Linkage

We linked data of participants in the *What Comes Next Cohort* to administrative databases at ICES to allow collection of additional variables. Based on postal codes obtained through the Registered Persons Database (RPDB), we categorized participants as living within urban (population ≥ 10,000) or rural (<10,000) regions and used census data to determine the median neighborhood household income level. The census-based Ontario Marginalization Index was used to estimate degree of marginalization [16].

We obtained data on cancer history through linkage with the Ontario Cancer Registry (OCR), which collects data on all incident invasive cancers, excluding non-melanoma skin cancers, since 1964 [17].

### 2.4. Data Analysis

We report baseline characteristics at the time of first genetic test. Continuous data are reported as mean (SD) or median (IQR) and categorical data as frequencies and percentages. Groups were compared using Mann–Whitney U tests and chi-squared tests. For continuous variables, temporal trends were evaluated using linear regression, with year of testing as the single covariate. For count variables, temporal trends were analyzed using Poisson regression with year of testing as the covariate; robust standard errors were used to manage mild overdispersion [18]. The Cochrane Armitage test for trend was used for proportional data. All reported *p*-values are two-sided. Analyses were performed using R, version 3.3. In accordance with ICES policies, we suppressed cells with < 6 individuals.

## 3. Results

### 3.1. Cohort Characteristics

A total of 15,986 women underwent *BRCA1* and/or *BRCA2* testing during the study period, including 13,619 (85.2%) women who underwent non-predictive testing and 2367 (14.8%) women who underwent predictive testing (Table 1). Median age was 53 years (IQR 43–63) and was significantly lower among women who underwent predictive testing (median 44, IQR 33–57) than among women who underwent non-predictive testing (median 54, IQR 45–64; *p* < 0.001). Most women tested were of European ancestry (63.4%); women of Ashkenazi Jewish ancestry accounted for 19.6% of women undergoing non-predictive testing (*n* = 2663) and 10.9% (*n* = 283) of women undergoing predictive testing (*p* < 0.001). Among women who underwent predictive testing, 40.4% (*n* = 957) tested positive for a pathogenic/likely pathogenic variant. Positive test results were reported in 7.9% (*n* = 1077) of women who underwent non-predictive testing.

### 3.2. Test Utilization

**Figure 1:**
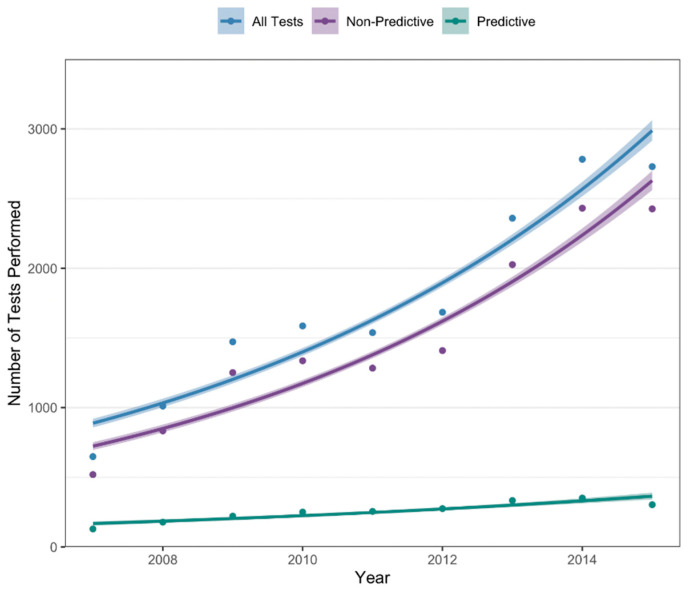
Annual number of tests performed from 2007–2015.

The number of tests performed in each full year of the study period is shown in Figure 1. Overall, there was a significant increase in the number of tests performed annually (*p* < 0.001), from 648 tests performed in 2007 to 2729 tests performed in 2015. This trend was seen for both predictive (129 tests in 2007; 303 tests in 2015; *p* < 0.001) and non-predictive testing (519 tests in 2007; 2426 tests in 2015, *p* < 0.001).

### 3.3. Age at Testing

**Figure 2:**
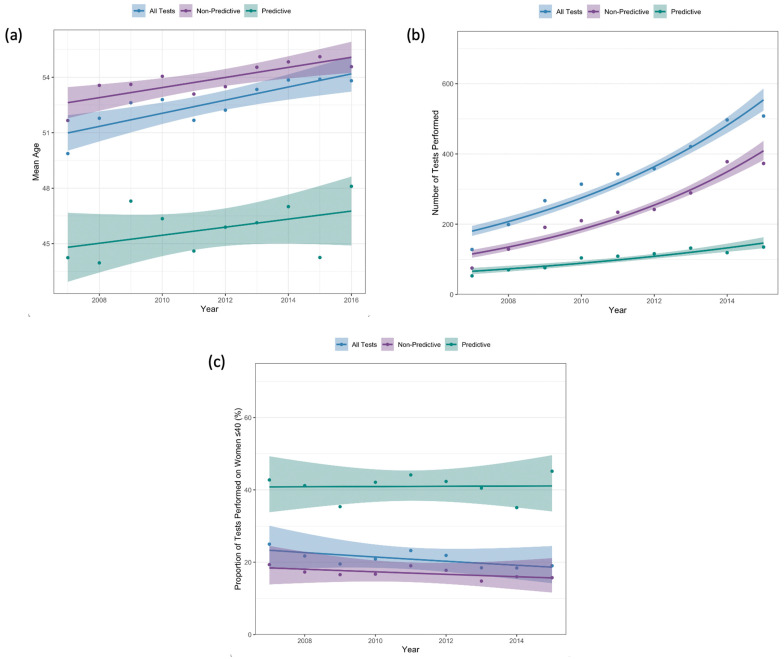
(**a**) Mean age at first test; (**b**) number of tests performed on women ≤ 40 years old; (**c**) proportion of first tests performed on women ≤ 40 years old.

Figure 2a demonstrates the mean age of women at first test for each year of the study period. Among all women tested, mean age at first test increased from 49.9 years (SD 13.8) in 2007 to 53.8 years (SD 13.7) in 2016 (*p_trend_* < 0.001). Although the number of tests performed on women ≤ 40 years also increased, from 128 tests in 2007 to 508 tests in 2015 (*p_trend_* < 0.001, Figure 2b), the proportion of all tests performed on women ≤40 years decreased, from 25.0% in 2007 to 19.0% in 2015 (*p_trend_* < 0.001; Figure 2c).

For women who underwent predictive testing, there was no statistically significant change in mean age at first test from 2007 (mean 44.2 years, SD 15.1) to 2016 (mean 48.1, SD 17.6) (*p_trend_* = 0.35) (Figure 2a). Although there was a significant increase in the number of tests performed on women ≤40 years, from 53 tests in 2007 to 135 tests in 2015 (*p_trend_* < 0.001), there was no significant change in the proportion of all predictive tests that were performed on women ≤40 years (42.7% in 2007 to 45.2% in 2015; *p_trend_* = 0.93; Figure 2c). As a proportion of all tests performed, predictive tests on women ≤40 significantly decreased from 10.4% in 2007 to 5.1% in 2015 (*p_trend_* < 0.001).

### 3.4. Cancer History

**Figure 3:**
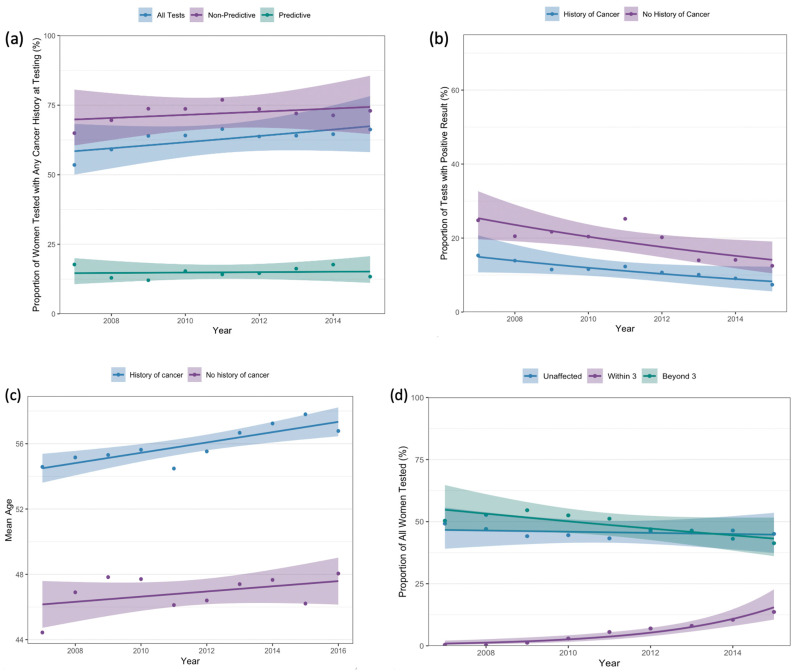
(**a**) Proportion of tests performed on women with a history of any cancer prior to testing, (**b**) proportion of positive tests by cancer history, (**c**) mean age at first test by cancer history, (**d**) proportion of tests performed by timing of breast cancer diagnosis.

A total of 10,262 (64.2%) women had a history of any cancer at the time of testing. The proportion of women with a personal cancer history increased from 53.5% in 2007 to 66.3% in 2015 (*p_trend_* < 0.001) (Figure 3a). Among women who underwent predictive testing, there was no significant change in the proportion with a history of cancer at testing (17.7% in 2007, 13.4% in 2015; *p_trend_* = 0.56). When stratified by personal cancer history, the proportion of women who tested positive for pathogenic/likely pathogenic variants decreased from 24.8% in 2007 to 12.5% in 2015 among women without a cancer diagnosis at testing (*p_trend_* < 0.001) and decreased from 15.3% to 7.4% among women with a cancer history at time of testing (*p_trend_* < 0.001; Figure 3b).

Among women with a history of cancer prior to testing, mean age at the time of testing increased from 54.6 years (SD 12.7) in 2007 to 56.8 years (SD 13.1) in 2016 (*p_trend_* = 0.002). Among those without a history of cancer prior to testing, there was a non-statistically significant increase in age at testing from 2007 (mean 44.4 years, SD 12.9) to 2016 (mean 48.1 years, SD 13.0) (*p_trend_* = 0.21) (Figure 3c).

A total of 8727 women had a history of breast cancer at the time of testing. Figure 3d demonstrates timing of breast cancer diagnosis in relation to testing. Although, among the entire cohort, the proportion of women without a breast cancer diagnosis at testing did not change over time (49.2% in 2007, 45.1% in 2015; *p_trend_* = 0.90), women who underwent genetic testing within 3 months of breast cancer diagnosis as a proportion of all women tested increased from 0.39% in 2007 to 13.6% in 2015 (*p_trend_* < 0.001). In 2015, 24.8% of women with a history of breast cancer at testing were tested within 3 months of cancer diagnosis, as compared to 0.77% in 2007.

## 4. Discussion

In this study of 15,986 women who underwent *BRCA1* and/or *BRCA2* testing after the introduction of a publicly funded program, use of testing increased annually. However, we identified several shortcomings in testing. The mean age at testing increased over the study period and women ≤ 40 years accounted for decreasing proportions of those tested. Although women with breast cancer were increasingly being tested around the time of cancer diagnosis—when test results can inform treatments—proportionally fewer tests over time were performed on women prior to cancer development, when risk-reduction strategies would be most effective.

Large-scale genetic testing programs, such as those for hereditary breast and ovarian cancer syndromes, typically have two broad aims: (1) identify pathogenic variants among individuals with cancer to inform treatment selection; (2) identify carriers prior to cancer development who would be eligible for risk-reduction. With respect to the first aim, under-utilization of genetic testing among women with breast cancer is a known phenomenon. In the US, only 20–30% of eligible individuals with cancer undergo testing [19]. This deficiency is attributed to issues including insufficient referral, limited access, and inadequate follow-through by patients [19]. In a Kaiser Permanente Washington health system study, a large proportion of eligible women with insurance coverage and access to specialty genetic services did not undergo testing [5]. In our study of a publicly funded testing program in Ontario, Canada, where cost was similarly not an issue, we found increasing utilization annually, suggesting greater awareness of and access to testing over time. Importantly, not only did women with cancer account for greater proportions of those being tested across the study period, but testing was also increasingly occurring around the time of cancer diagnosis, rather than remote from diagnosis. This is critical as knowledge of variant status in proximity to diagnosis allows this information to be utilized when selecting treatments and creates opportunities to discuss prophylactic strategies.

While a publicly funded testing program appears to address the first aim of large-scale testing programs, we found major gaps in achieving the second aim of identifying pathogenic variant carriers prior to cancer development. Arguably, the greatest opportunity for health system impact is in targeting unaffected individuals. In the US, it is estimated that only 10% of those without a history of breast cancer who are *BRCA1* or *BRCA2* carriers have been identified and these unaffected women account for approximately 220,000 of the over 348,000 variant carriers [20]. Women who carry *BRCA1* and/or *BRCA2* pathogenic variants develop breast cancer at a mean age of 45 [1]. Concerningly, our study demonstrates that nearly 10 years after inception of a publicly funded testing program, mean age at testing was 54 years and was increasing. Resources for testing and counseling must inherently be balanced between women with and without cancer; however, the prioritization of women with cancer for genetic testing suggests a major lost opportunity in identifying high-risk women who are cancer-free.

The two major factors dictating the proportion of cancer-free women undergoing testing are testing criteria and frequency of cascade testing. The eligibility criteria set by the Ontario Ministry of Health are heavily reliant on personal cancer history. At the time of this study, those without a cancer history were only eligible for testing if they had a relative known to carry a pathogenic variant, were Ashkenazi Jewish and had a strongly suggestive family history, or had a pathogenic variant carrier risk estimated at >10% by established risk calculators [14]. In our study, 12.5% of women without a cancer history who underwent testing carried a pathogenic variant, suggesting underutilization of testing among these women. Although family history can successfully identify higher-risk individuals, ascertainment is not infallible. A study of US primary care physicians (PCPs) demonstrated shortcomings in the ability of PCPs to accurately ascertain family history [21]. Reliance on family history, as is the case in Ontario as well as in National Comprehensive Cancer Network (NCCN) [22] and US Preventive Services Task Force (USPSTF) [23] guidelines, likely misses large numbers of carriers who would benefit from testing. Broader eligibility criteria, specifically for those who are cancer-free, could capture high-risk individuals missed by current testing standards.

As one example of broadening testing criteria, various groups have investigated the utility of population-based testing. In an Israeli study of population-based testing of Ashkenazi Jewish women, 51% of women identified as *BRCA1* or *BRCA2* mutation carriers did not have a family history indicating need for testing [24]. In a separate randomized trial of population-based testing of Ashkenazi Jewish women, 60% of identified carriers did not meet family history criteria for testing; population-based testing was estimated to increase carrier identification by up to 150% [25,26]. A Canadian study further found a 6% pathogenic variant rate when otherwise unselected women diagnosed with breast cancer underwent rapid genetic testing [27]. Challenges of population-based testing include cost and access to genetic counseling/testing. However, innovative strategies can address these goals. For example, the Canadian Screen Project [28] allowed any adult Canadian to self-refer for *BRCA1* and *BRCA2* testing via mailed saliva kits. Over 2 years, 1269 women were tested, only 3.4% of whom had a personal history of breast cancer. 2.4% of those tested carried a pathogenic variant. Genetic counseling, which was offered at the discretion of test-negative participants, was utilized by only 5% of these individuals. Programs such as these demonstrate how direct-to-consumer models can complement established programs to increase testing among those not meeting stringent criteria.

Identifying carriers prior to cancer development also depends on effective cascade testing. Assuming genetic testing at the time of cancer diagnosis coupled with cascade testing of 70% of at-risk relatives, it is estimated that the 3.9 million individuals in the US carrying pathogenic variants in 18 cancer susceptibility genes could be identified in < 10 years [29]. While previous studies demonstrate that > 95% of pathogenic variant carriers communicate their status to at least one relative [30,31], only 30–60% of relatives proceed to cascade testing [32,33,34,35]. Cited barriers include fear of test results, concerns about genetic discrimination, limited knowledge of the impact of the proband’s variant status on the relative’s health, and lack of access to genetics referral [34,36,37]. Multiple solutions have been explored to address this. Among the most effective is healthcare personnel, specifically psychosocial worker or counselor, involvement in discussions with family members, as well as clinician-mediated direct relative contact [37,38]; the latter was shown in meta-analysis to increase test uptake by at-risk relatives from 35% to 63% [38].

This study evaluates utilization of *BRCA1*/*BRCA2* testing in a near population-based cohort over a 10-year period in a single-payer publicly funded healthcare system, where cost is not a barrier to testing. Still, limitations exist. Specific to this study, we cannot identify individuals of a single family who underwent testing, limiting our ability to estimate use of cascade testing. However, inferences can be made based on utilization of predictive testing. Additionally, although we captured approximately 70% of testing in Ontario, regions not included in this study may demonstrate disparate patterns of testing based on access and socioeconomic factors, limiting generalizability. Finally, increasingly, various jurisdictions have shifted toward genetic panel testing, which is not captured in this study; however, we would expect similar patterns in use of single gene versus panel testing. Future studies of testing trends among individuals undergoing panel testing and an exploration of the barriers to testing among high-risk, cancer-free individuals are needed.

## 5. Conclusions

Genetic testing is a powerful tool for assessing inherited cancer susceptibility, enabling individuals and their healthcare providers to make informed decisions about risk management, prevention, and early detection strategies. The importance of genetic testing extends beyond individual health, also facilitating a broader understanding of familial cancer risks, aiding in development of comprehensive strategies to mitigate hereditary cancer burden within families. Our study demonstrated that a publicly funded genetic testing program can be successfully utilized to identify individuals with pathogenic variants near the time of cancer diagnosis, when this information can inform treatment decision-making. However, to truly reduce cancer burden more broadly, efforts need to shift toward utilizing such programs to identify individuals prior to cancer development. We found significant shortcomings in the current testing paradigm, whereby women who are cancer-free but still at high-risk of carrying pathogenic variants—a population who arguably stands to gain the most from testing—demonstrate low test uptake. To have real impact on cancer prevention, strategies focused on increasing genetic test utilization prior to cancer development, including by broadening testing criteria and targeting individuals eligible for cascade testing, require prioritization and implementation.

## Figures and Tables

**Table 1:** Cohort characteristics at date of first test.

Characteristic		All Participants (n = 15,986)	Non-Predictive Testing (n = 13,619, 85.2%)	Predictive Testing (n = 2367, 14.8%)	*p*-Value
Age at first test (median, IQR)		53 (43–63)	54 (45–64)	44 (33–57)	<0.001
Ancestry (%)					
	European	10,130 (63.4)	8519 (62.6)	1611 (68.1)	<0.001
	Southeast Asian	1048 (6.6)	952 (7.0)	96 (4.1)	<0.001
	Central/South Asian or Middle Eastern	1282 (8.0)	1114 (8.2)	168 (7.1)	0.08
	African/Caribbean	606 (3.8)	522 (3.8)	84 (3.5)	0.54
	Latin/Hispanic	495 (3.1)	449 (3.3)	46 (1.9)	0.001
	Other	880 (5.5)	681 (5.0)	199 (8.4)	<0.001
	Unknown	3023 (18.9)	2599 (19.1)	424 (17.9)	0.19
Ashkenazi Jewish (%)		2946 (18.4)	2663 (19.6)	283 (10.9)	<0.001
Neighborhood income quintile (%)					<0.001
	Rural	1340 (8.4)	1083 (8.0)	257 (10.9)	
	Urban, lowest quintile	1940 (12.1)	1657 (12.2)	283 (12.0)	
	Urban, second quintile	2378 (14.9)	2046 (15.0)	332 (14.0)	
	Urban, third quintile	2522 (15.8)	2136 (15.7)	386 (16.3)	
	Urban, fourth quintile	3298 (20.6)	2811 (20.6)	487 (20.6)	
	Urban, highest quintile	4481 (28.0)	3864 (28.4)	617 (26.1)	
Marginalization summary score (mean, SD)		3.03 (0.77)	3.03 (0.77)	2.99 (0.75)	0.01
Year of first test (%)					<0.001
	2007–2009	2933 (18.3)	2407 (17.7)	526 (22.2)	
	2010–2012	4616 (28.9)	3845 (28.2)	771 (32.6)	
	2013–2016	8437 (52.8)	7367 (54.1)	1070 (45.2)	
Result of first test (%)					<0.001
	Positive	2034 (12.7)	1077 (7.9)	957 (40.4)	
	Variant of uncertain significance	1166 (7.3)	1134 (8.3)	32 (1.4) *	
	Negative	11,408 (71.4)	11,408 (83.8)	0 (0)	
	Predictive Negative	1377 (8.6)	0 (0)	1377 (58.2)	

* Women who underwent predictive testing for a familial VUS.

## Data Availability

The dataset from this study is held securely in coded form at ICES. While data sharing agreements prohibit ICES from making the dataset publicly available, access may be granted to those who meet pre-specified criteria for confidential access, available at www.ices.on.ca/DAS (accessed on 19 June 2025). The full dataset creation plan and underlying analytic code are available from the authors upon request, understanding that the computer programs may rely upon coding templates or macros that are unique to ICES and therefore either inaccessible or may require modification.
